# Analysis of characteristics and forecast of unintentional injury deaths of children under age 5 from 2013 to 2019 in Sichuan, China

**DOI:** 10.1186/s12889-022-14600-z

**Published:** 2022-11-21

**Authors:** Jinnuo Hu, Min Luo, Linkun He, Ziling Zhao

**Affiliations:** Department of Pediatrics, Sichuan Provincial Hospital for Women and Children (Affiliated Women and Children’s Hospital of Chengdu Medical College), Chengdu, China

**Keywords:** Unintentional injury death, Children under five years old, Characteristic, Ethnic, Rural, ARIMA

## Abstract

**Objective:**

Through the study of death characteristics and trend prediction, it is hoped that key populations, regions and seasons can be identified, thereby providing evidence support for the efficient prevention and control management of unintentional injury deaths.

**Method:**

We collected information on 8630 unintentional deaths of children under age 5 from local surveillance systems, analyzed by chi-square test and predicted by the seasonal ARIMA model.

**Results:**

About 33.1% of child deaths were under the age of 1, 60.5% were boys, 37.6% were in urban areas, 2.6% were among ethnic Tibetans, 6.8% were among ethnic Yi, and 46.6% died inside houses. The top three of total deaths were accidental drowning (35.0%), accidental suffocation (32.7%) and traffic accident (15.5%). The ratio of males to females in traffic accidents (1.28:1) and poisoning (1.30:1) deaths was relatively lower than accidental falls (1.62:1) and drowning (1.85:1). The causes of death ratio in rural and urban areas were: drowning (1.83:1), poisoning (1.75:1), suffocation (1.62:1), traffic (1.41:1), and falling (1.24:1). Children's deaths of ethnic minority groups of Tibetan and Yi increased year by year (*χ*^2^=75.261, *P*< 0.001). Tibetan and Yi groups had the most deaths in summer, and Han in winter (*χ*^2^=29.093, *P*< 0.001). Accidental suffocation accounted for 78.2 percent of the total unintentional deaths of children under age 1. And drowning accounted for only 2.4 percent. The model SERIMA (1, 1, 2) (2, 0, 0) [12] is suitable for describing and predicting unintentional injury deaths of children under age 5.

**Conclusion:**

We should combine death surveillance with qualitative investigation or in-depth quantitative investigation to further analyze unintentional injury deaths in children.

## Key messages

**What is already known on this topic**

Most child deaths occur in children younger than 5 years of age, and unintentional injuries are the leading cause of death, mainly in low- or middle-income areas. ​Sichuan is one of China's most impoverished provinces, with a high number of child injury deaths each year.

**What this study adds**

Children's deaths of ethnic minority groups of Tibetan and "Yi" increased as years went (*χ*^2^ = 75.261, *P* < 0.001). The model SERIMA (1, 1, 2) (2, 0, 0) [12] is suitable for describing and predicting unintentional injury deaths of children under age 5.

**How this study might affect research, practice or policy**

This study will make up for the lack of comprehensive analysis and visualization of the characteristics of unintentional injury deaths among children in Sichuan and provides evidence-based support for efficient prevention and control management of unintentional injury deaths.

## Introduction

More than 5 million children die every year around the world, and more than 80% of those aged under 5 [1]. Unintentional injuries are the main causes of children's death, resulting in more than half deaths of children in some countries[2]. As the leading killer of child deaths [3], unintentional injuries are more common in less developed regions. And according to WHO, more than 80% of the unintentional injuries occurred in low or middle-income countries [4]. As one of the largest developing countries in the world, China more than 200,000 child deaths each year as a result of unintentional injuries. And as one of China's most impoverished provinces, Sichuan nearly has the country's highest child mortality rate.

There have been numerous studies about injury deaths of children under age 5 around the world [5–23]. The characteristics of unintentional injury deaths in children in these studies vary considerably across populations, regions and time periods. Besides, studies in Sichuan are not comprehensive or sufficient and need updating [6].

Reducing children's deaths is part of the Millennium Development Goals (MDG) [7]. The United Nations Sustainable Development Goals propose that preventable deaths of children under 5 years of age should be eliminated by 2030, and every country should strive to reduce the mortality rate to less than 25‰ of children under 5 [8]. Reducing unintentional injury deaths in children, is vital for reducing child mortality.

This study intend to study temporal, regional, and demographic distributional characteristics and use scientific methods to predict trends, which will make up for the lack of comprehensive analysis and visualization of the characteristics of unintentional injury deaths among children in Sichuan.

## Objectives

Through analysis of the information in Sichuan, this study hopes to find out the epidemiological characteristics of unintentional injury deaths in local children under age 5, to identify the focus of injury prevention in people of different nationalities, genders, ages and etc., and to make scientific prediction of short-term death trends. And it is hoped that key populations, key regions and key seasons can be identified to provide evidence-based support for efficient prevention and control management of unintentional injury deaths.

## Methods

### Data sources

Data on child deaths in Sichuan Province from 2013 to 2019 used in this study came from the local maternal and child health surveillance system, which collects relevant data in accordance with Chinese regulations.

We applied for 8630 pieces of information of children died of unintentional injuries in all, including cities of death (a total of 21), the household registration (urban and rural), gender (male and female), ethnic gathering area (ethnic-minority areas of Tibetan and Yi and non-minority areas as Han group), the cause of death (drowning, suffocation, traffic accident, poisoning, falls, others), age (≥ 0 and < 1, ≥ 1and < 2, ≥ 2 and < 3, ≥ 3 and < 4, ≥ 4 and < 5), year of death (2013–2019), season (March–May as spring, June–August as summer, September–November as autumn, December-February as winter) and place of death (inside the house, in the hospital and others).

### Data analysis

The "hchinamap" package in RStudio v1.0.143 was used to map the number of accidental child deaths in Sichuan Province. Excel was used to draw circle diagram to reflect the composition of accidental death causes, and the composition ratio of the top four causes was marked. The chi-square test was used to compare the epidemiological characteristics of unintentional deaths by gender, region and ethnicity. The seasonal ARIMA model is used to predict the future trend of total injury deaths per month. *P* < 0.05 is considered statistically significant.

## Results

### Causes of death by region

​The unintentional injury deaths of children under the age of five in Sichuan are mainly in the more densely populated eastern regions. Chengdu, the capital city of Sichuan, had the most children's deaths (915). Western areas with higher elevations and lower population densities had the least deaths, such as Ganzi city (108) and Aba city (126).

**Fig. 1:**
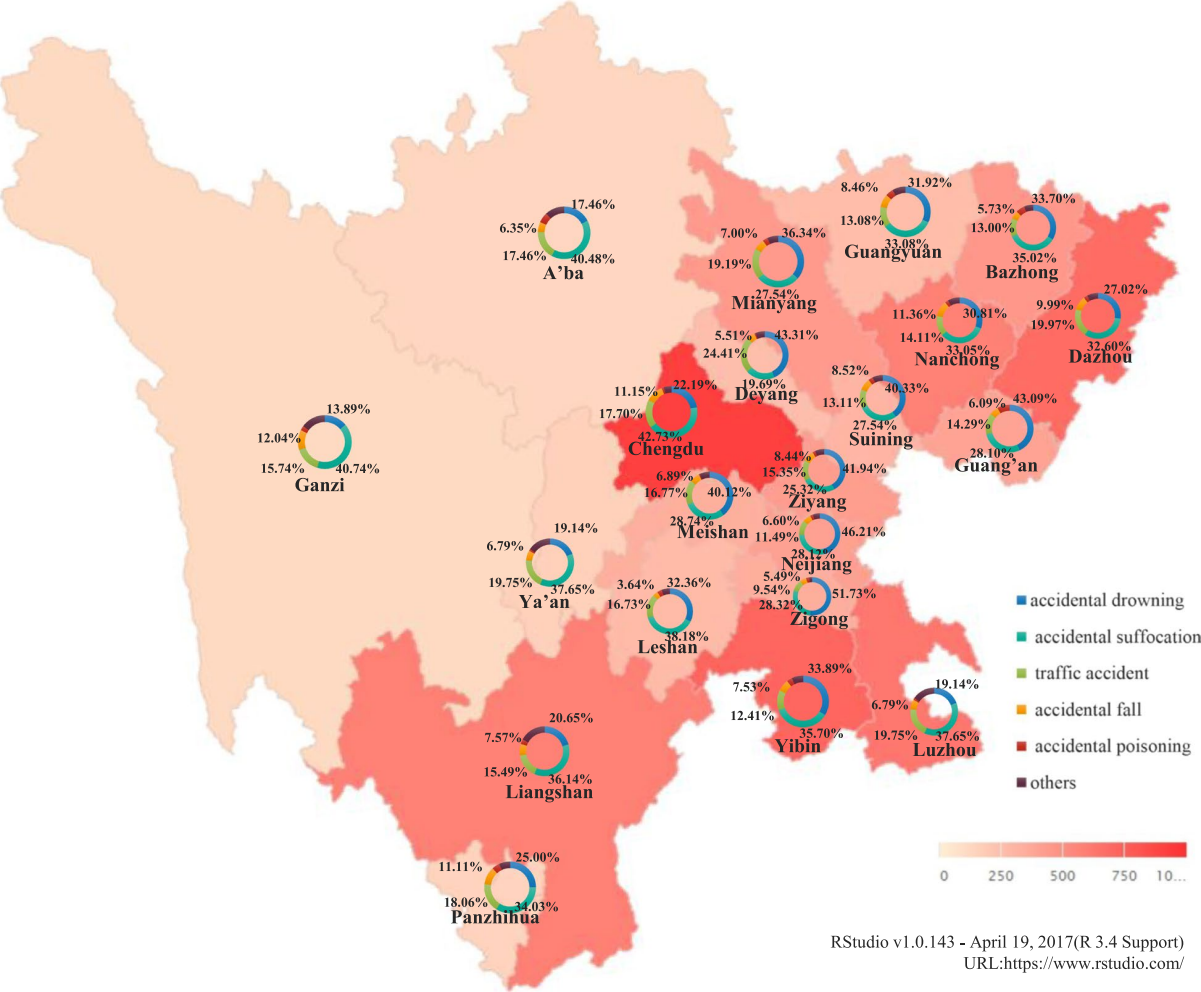
Proportion of main causes of deaths in different cities

The leading causes of unintentional injury deaths in virtually every city are suffocation and drowning. ​Areas from northern Mianyang city (36.3%) to southern Zigong city (51.7%) and eastern Guangyuan city (31.9%) to western Meishan city (40.1%) mainly had drowning deaths. The numerous rivers in these above-mentioned areas create additional conditions for drowning. Ganzi (40.7%), Aba (40.5%) and Liangshan (36.1%) in the western areas are gathering places of ethnic minority groups with Tibetans and Yi people, where children are mainly killed by accidental suffocation. Details can be seen in Fig. 1.

### Trends of different causes of death

**Fig. 2:**
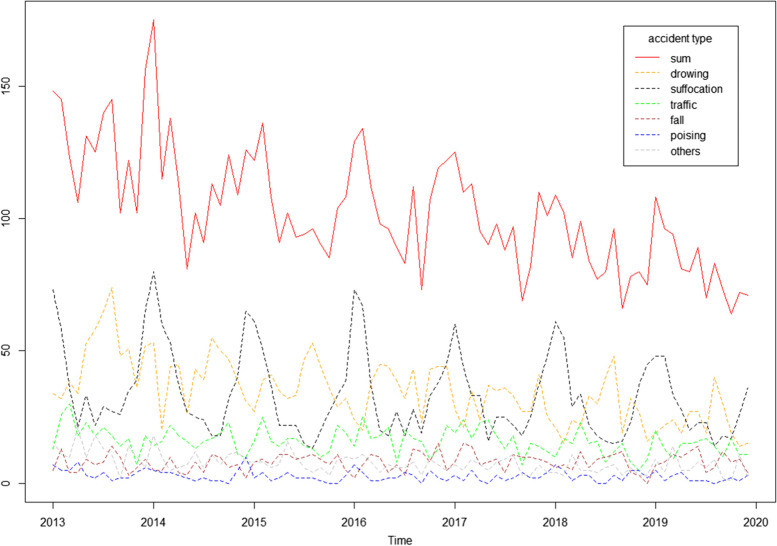
*Trends of main causes of deaths by month from 2013 to 2019.*The dots on the abscissa represent the January of the current year, and the regions before the next year represent the January to December of the current year. For example, 2013 represents January 2013 and 2014 represents January 2014

Total unintentional injury deaths among children under the age of five in Sichuan have been decreasing year on year from 2013 to 2019. Total deaths happened higher in cold weather (around January). Suffocation deaths occur significantly more commonly in winter and drowning deaths occur more generally in summer. Suffocation and drowning deaths are both on a year-on-year downward trend. However, other causes of death do not fluctuate seasonally, and their long-term trends are relatively stable. Details can be seen in Fig. 2 and Table 1.

**Table 1:** Total child deaths per month, 2013–2019

Variables	2013	2014	2015	2016	2017	2018	2019
January	148	175	122	129	125	109	108
February	145	115	136	134	110	102	96
March	123	138	108	112	113	85	94
April	106	113	91	98	95	99	81
May	131	81	102	96	90	84	80
June	125	102	93	89	98	77	89
July	140	91	94	83	88	80	70
August	145	113	96	112	97	96	83
September	102	105	90	73	69	66	73
October	122	124	85	107	82	78	64
November	102	109	104	119	110	80	72
December	156	126	108	122	101	75	71
Sum	1545	1392	1229	1274	1178	1031	981

### Characteristics of unintentional deaths in children

Of the 8,360 unintentional injury deaths of children under the age of five in Sichuan from 2013 to 2019, 33.1% were under the age of one, 60.5% were boys, 37.6% were in rural areas, 2.6% were ethnic Tibetan, and 6.8% were ethnic Yi. The main causes of total death were accidental drowning (35.0%), accidental suffocation (32.7%) and traffic accident (15.5%). A total of 46.6% of children died in the house and 26.0% in hospital.

### Comparison between different gender

About three-fifths of the children who die each year or season from unintentional injuries are boys. Nor does the ratio of boys to girls change by year or season. The proportion of male children's deaths increased with age (*χ*^2^ = 30.078, *P* < 0.001) among children who died unintentionally under 5 years old. There are significant gender differences in the ratio of different causes of death (*χ*^2^ = 42.077, *P* < 0.001). And the ratio of males to females in traffic accidents (1.28:1) and poisoning (1.30:1) deaths was relatively lower than accidental falls (1.62:1) and drowning (1.85:1).

### Comparison between rural and urban areas

In 2013, the number of urban deaths was approximately twice as high as in rural areas. And in 2019, the number of deaths in urban and rural areas was nearly the same. Urban areas have always had more deaths than rural areas, but the gap between them narrowed as the year goes (*χ*^2^ = 122.961, *P* < 0.001). Urban deaths are 1.5 times higher than rural deaths, regardless of season or age group. The causes of death in urban and rural areas were significantly different (*χ*^2^ = 40.571, *P* < 0.001). The top three causes of death in cities, from most to least, were drowning, suffocation and traffic. The top three causes of death in rural areas are: suffocation, drowning and traffic. The causes of death ratio in urban and rural areas were: drowning (1.83:1), poisoning (1.75:1), suffocation (1.62:1), traffic (1.41:1), and falling (1.24:1). There are more deaths at home in rural areas and fewer in hospitals than in urban areas (*χ*^2^ = 43.550, *P* < 0.001).

### Comparison among different ethnic groups

The number of child deaths among ethnic Tibetan and Yi people has increased year on year. However, deaths of non-minority group Han decreased (*χ*^2^ = 75.261, *P* < 0.001). Tibetan and Yi groups had the most deaths in summer, and Han in winter (*χ*^2^ = 29.093, *P* < 0.001). Children under the age of one accounted for more than 40 percent of children under the age of five in the Tibetan and Yi ethnic-minority groups, and only about 30 percent in the Han non-minority group. (*χ*^2^ = 49.529, *P* < 0.001). There were statistically significant differences in the composition of causes of death among different ethnic groups (*χ*^2^ = 164.098, *P* < 0.001). The top three causes of death for Tibetans and Yi were: suffocation, drowning and traffic, while for Han: drowning, suffocation and traffic. Forty percent of the children died from accidental suffocation and about 17 percent from drowning among the ethnic Tibetan group, compared with 32 percent and 36 percent for the non-minority Han group. Tibetans and Yi people die more at home and less in hospitals than Han people (*χ*^2^ = 33.445, *P* < 0.001). Details can be seen in Table 2.

**Table 2:** Chi-square test of characteristics of unintentional injury deaths

Variables	Gender					Areas				
	Male	Percentage	Female	Percentage	M:F	Rural	Percentage	Urban	Percentage	R:U
Year of death										
2013	920	17.6%	625	18.3%	1.47:1	989	18.4%	556	17.1%	1.78:1
2014	828	15.9%	564	16.5%	1.47:1	929	17.3%	463	14.3%	2.01:1
2015	733	14.0%	496	14.5%	1.48:1	843	15.7%	386	11.9%	2.18:1
2016	806	15.4%	468	13.7%	1.72:1	847	15.7%	427	13.1%	1.98:1
2017	704	13.5%	474	13.9%	1.49:1	708	13.2%	470	14.5%	1.51:1
2018	635	12.2%	396	11.6%	1.60:1	559	10.4%	472	14.5%	1.18:1
2019	595	11.4%	386	11.3%	1.54:1	506	9.4%	475	14.6%	1.07:1
	*χ*^2^ = 6.436		*P* = 0.376			*χ*^2^ = 122.961		*P* < 0.001*		
Season of death										
Spring	1300	24.9%	820	24.1%	1.59:1	1357	25.2%	763	23.5%	1.78:1
Summer	1226	23.5%	835	24.5%	1.47:1	1271	23.6%	790	24.3%	1.61:1
Autumn	1195	22.9%	741	21.7%	1.61:1	1184	22.0%	752	23.1%	1.57:1
Winter	1500	28.7%	1013	29.7%	1.48:1	1569	29.2%	944	29.1%	1.66:1
	*χ*^2^ = 3.392		*P* = 0.335			*χ*^2^ = 4.076		*P* = 0.253		
Age of death										
[0,1)	1629	31.2%	1224	35.9%	1.33:1	1773	32.9%	1080	33.2%	1.64:1
[1,2)	1130	21.6%	762	22.4%	1.48:1	1192	22.2%	700	21.5%	1.70:1
[2,3)	1135	21.7%	693	20.3%	1.64:1	1123	20.9%	705	21.7%	1.59:1
[3,4)	771	14.8%	424	12.4%	1.82:1	756	14.0%	439	13.5%	1.72:1
[4,5)	556	10.6%	306	9.0%	1.82:1	537	10.0%	325	10.0%	1.65:1
	*χ*^2^ = 30.078		*P* < 0.001*			*χ*^2^ = 1.474		*P* = 0.831		
Causes of death										
Accidental fall	417	8.0%	258	7.6%	1.62:1	374	7.0%	301	9.3%	1.24:1
Accidental poisoning	129	2.5%	99	2.9%	1.30:1	145	2.7%	83	2.6%	1.75:1
Accidental suffocation	1643	31.5%	1176	34.5%	1.40:1	1745	32.4%	1074	33.1%	1.62:1
Traffic accident	752	14.4%	586	17.2%	1.28:1	783	14.6%	555	17.1%	1.41:1
Accidental drowning	1898	36.4%	1027	30.1%	1.85:1	1890	35.1%	1035	31.9%	1.83:1
Others	382	7.3%	263	7.7%	1.45:1	444	8.3%	201	6.2%	2.21:1
	*χ*^2^ = 42.077		*P* < 0.001*			*χ*^2^ = 40.571		*P* < 0.001*		
Places of deaths										
Inside the house	2432	46.6%	1591	46.7%	1.53:1	2618	48.7%	1405	43.2%	1.86:1
In the hospital	1231	23.6%	836	24.5%	1.47:1	1167	21.7%	900	27.7%	1.30:1
Others	1558	29.8%	982	28.8%	1.59:1	1596	29.7%	944	29.1%	1.69:1
	*χ*^2^ = 1.524		*P* = 0.467			*χ*^2^ = 43.550		*P* < 0.001*		
Sum	5221	60.5%	3409	39.5%		5381	62.4%	3249	37.6%	

Variables	Ethnics							Sum	
	Tibetan	Percentage	Yi	Percentage	Han	Percentage	T: Y: H		
Year of death									
2013	33	14.5%	72	12.3%	1440	18.4%	0.02: 0.05:1	1545	17.9%
2014	34	14.9%	55	9.4%	1303	16.7%	0.03: 0.04:1	1392	16.1%
2015	31	13.6%	71	12.1%	1127	14.4%	0.03: 0.06:1	1229	14.2%
2016	23	10.1%	110	18.8%	1141	14.6%	0.02: 0.10:1	1274	14.8%
2017	33	14.5%	100	17.1%	1045	13.4%	0.03: 0.10:1	1178	13.7%
2018	31	13.6%	92	15.7%	908	11.6%	0.03: 0.10:1	1031	11.9%
2019	43	18.9%	86	14.7%	852	10.9%	0.05: 0.10:1	981	11.4%
	*χ*^2^ = 75.261			*P* < 0.001*					
Season of death									
Spring	60	26.3%	153	26.1%	1907	24.4%	0.03: 0.08:1	2120	24.6%
Summer	74	32.5%	165	28.2%	1822	23.3%	0.04: 0.09:1	2061	23.9%
Autumn	45	19.7%	136	23.2%	1755	22.5%	0.03: 0.08:1	1936	22.4%
Winter	49	21.5%	132	22.5%	2332	29.8%	0.02: 0.06:1	2513	29.1%
	*χ*^2^ = 29.093			*P* < 0.001*					
Age of death									
[0,1)	94	41.2%	252	43.0%	2507	32.1%	0.04: 0.10:1	2853	33.1%
[1,2)	40	17.5%	96	16.4%	1756	22.5%	0.02: 0.05:1	1892	21.9%
[2,3)	47	20.6%	90	15.4%	1691	21.6%	0.03: 0.05:1	1828	21.2%
[3,4)	26	11.4%	78	13.3%	1091	14.0%	0.02: 0.07:1	1195	13.8%
[4,5)	21	9.2%	70	11.9%	771	9.9%	0.03: 0.09:1	862	10.0%
	*χ*^2^ = 49.529			*P* < 0.001*					
Causes of death									
Accidental fall	24	10.5%	45	7.7%	606	7.8%	0.04: 0.07:1	675	7.8%
Accidental poisoning	10	4.4%	18	3.1%	200	2.6%	0.05: 0.09:1	228	2.6%
Accidental suffocation	92	40.4%	214	36.5%	2513	32.2%	0.04: 0.09:1	2819	32.7%
Traffic accident	35	15.4%	92	15.7%	1211	15.5%	0.03: 0.08:1	1338	15.5%
Accidental drowning	38	16.7%	116	19.8%	2771	35.5%	0.01: 0.04:1	2925	33.9%
Others	29	12.7%	101	17.2%	515	6.6%	0.06: 0.20:1	645	7.5%
	*χ*^2^ = 164.098			*P* < 0.001*					
Places of deaths									
Inside the house	117	51.3%	326	55.6%	3580	45.8%	0.03: 0.09:1	4023	46.6%
In the hospital	40	17.5%	95	16.2%	1932	24.7%	0.02: 0.05:1	2067	24.0%
Others	71	31.1%	165	28.2%	2304	29.5%	0.03: 0.07:1	2540	29.4%
	*χ*^2^ = 33.445			*P* < 0.001*					
Sum	228	2.6%	586	6.8%	7816	90.6%		8630	

^*^Ethnics is defined in terms of agglomeration areas

### Comparison among different age groups

**Fig. 3:**
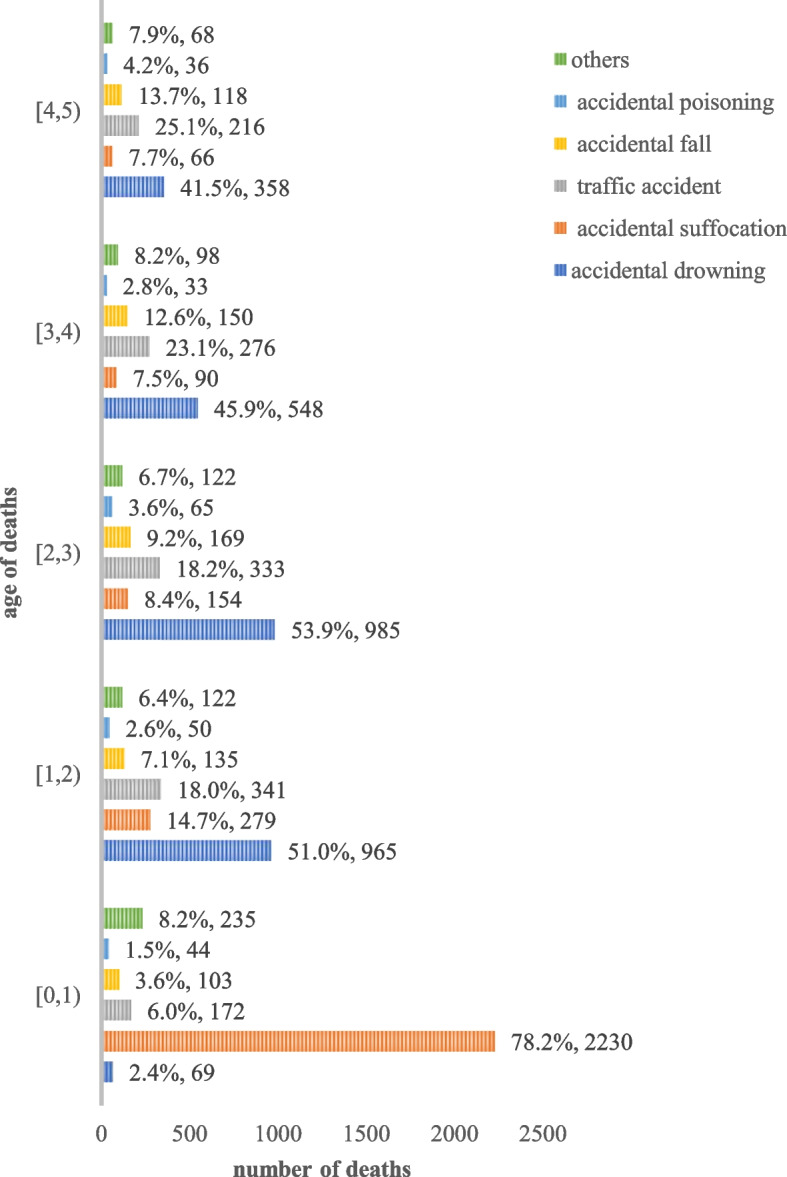
Causes of deaths of children under 5 years old by age

Accidental suffocation accounted for 78.2 percent of the total unintentional deaths of children under age 1. Drowning accounted for only 2.4 percent of deaths. Among unintentional deaths in children older than 1, drowning accounted for half of the causes of death and accidental suffocation accounted for about 10 percent. Details can be seen in Fig. 3.

### Prediction of unintentional deaths in children

**Fig. 4:**
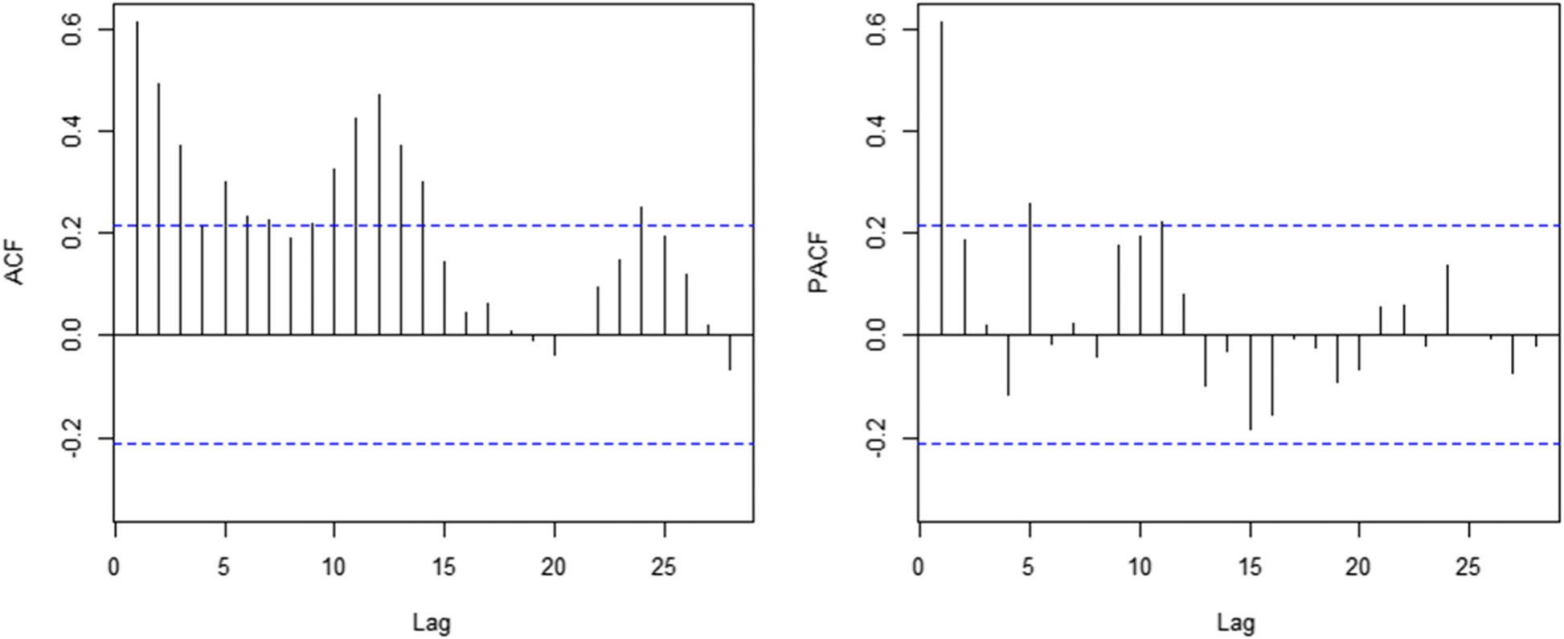
ACF and PACF plots of raw data

**Fig. 5:**
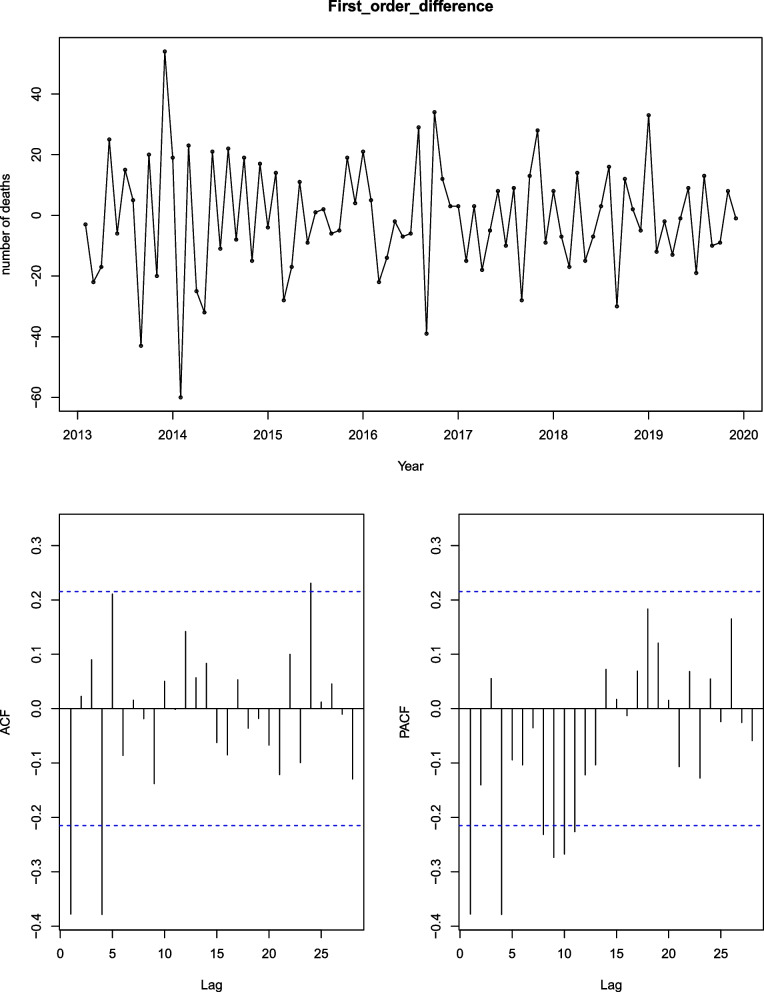
ACF and PACF plots of difference of raw data

**Fig. 6:**
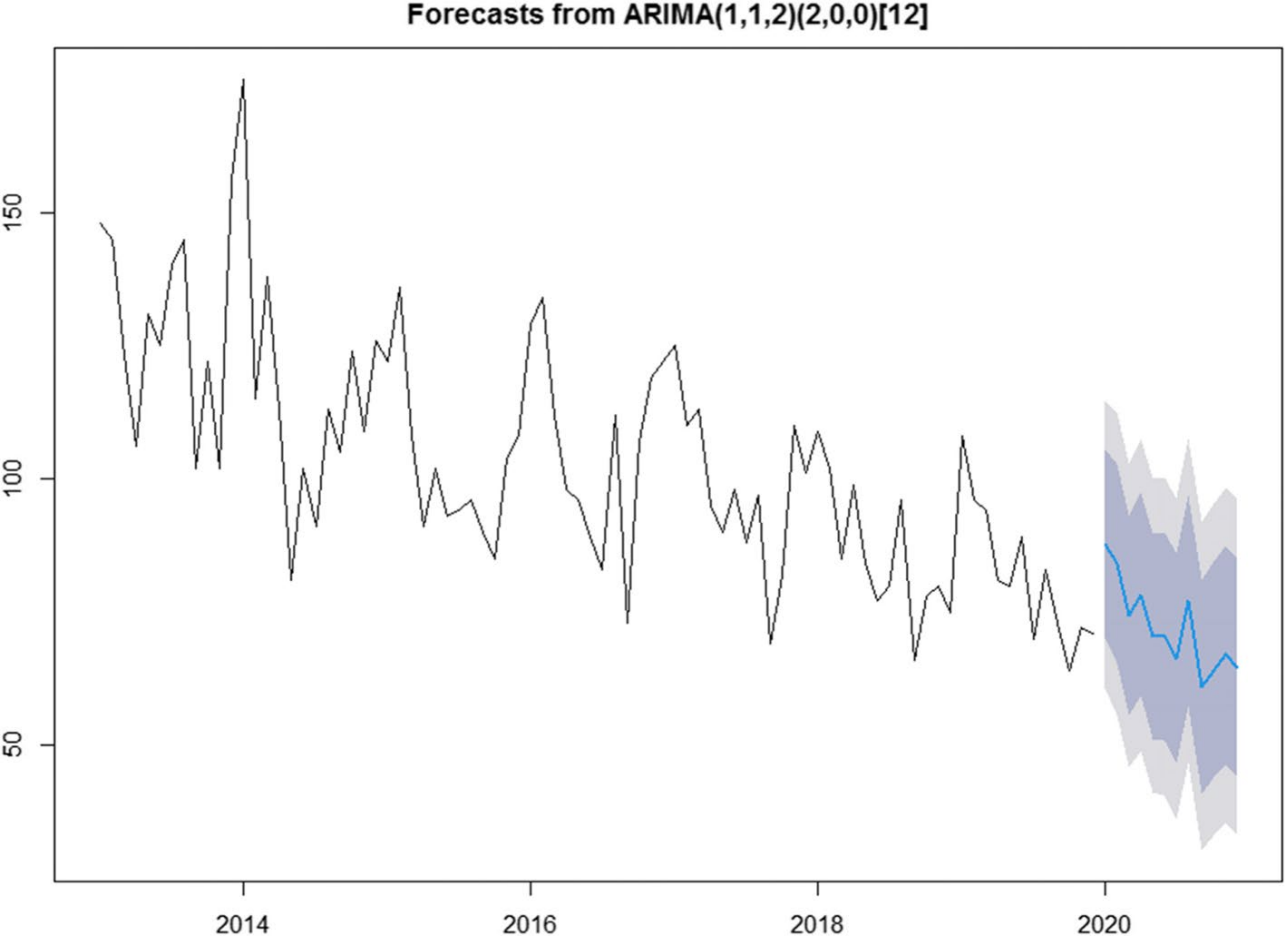
Prediction of unintentional injury deaths among children under 5 years old

We use time series to analyze the sum of unintentional injury deaths per month. The series is not white noise, with a downward trend and seasonal fluctuations, as can be seen in Fig. 2. Therefore, we use the seasonal ARIMA model to build the prediction model. On the basis of the ACF and PACF Figures (Fig. 4, Fig. 5) of the original series and difference series as references, the model SERIMA (1,1,2) (2,0,0) [12] is finally formed (details can be seen in Fig. 6) with model AICc = 599.81. The Box.test results show X-squared = 0.097078 with *p*-value = 0.7554 and the residuals are independent. The results of the Kolmogorov–Smirnov nonparametric test show that W = 0.99232 with *p*-value = 0.9062 and the residuals of the model are normally distributed.

## Discussion

Compared with a study of China 10 years ago [9] which showed that drowning and traffic accidents were the first two causes of total unintentional injury death, this study shows that leading causes are drowning (35%) and suffocation (32.7%). The result is different from that in Turkey [10] whose leading causes are traffic injuries (36.5%) and falls (12.0%) and in Pakistan [11] whose leading causes are drowning (22%), traffic injuries (12%). Results declared in this study that female children who died more and the death proportion of rural areas is higher are similar to that of many other China’s studies [12, 13], as well as to that of Japan [14] and Iran [15] in Asia, etc.. This study shows that the leading cause of unintentional death under age 1 was suffocation and the proportion of children injured to death under 1 year old was larger than other age groups, which is similar to that of Brazil [16] and the United States [17]. ​In the study, rural children accounted for the majority of deaths from each cause. A study in India [18] shows similar results. However, an Egyptian study [19] shows different results. More children die from unintentional injuries in rural areas than in urban areas. Given the relatively higher rates of intra-household deaths in rural and ethnic minority areas, it can be speculated that there may still be significant gaps in access to health services between urban and rural areas, and between different ethnic groups in Sichuan. Unintentional death differences exist in children of different ethics in America where non-Hispanic black children died more compared with non-Hispanic white and Hispanic children [20, 21]. Results that children in ethnic minority groups are more inclined to die of unintentional injuries appear in Bernard SJ [22] and Gilchrist J's [23] studies where American Indian/Alaska Natives (AI/ANs) and blacks had consistently more total injury death than the White. In this study, children of ethnic groups “Yi” and “Tibetan” died more compared with the largest ethnic group “Han” in China.

In recent years, the total number of unintentional injury child deaths in Sichuan has dropped significantly. Drowning and suffocation deaths declined the most, which may be related to previously higher numbers of drowning and suffocation deaths, suggesting that prevention and control of the leading cause of death has achieved great results. However, their rate of decline is becoming slower and seasonal fluctuations remain. Suffocation was the leading cause of death for both rural areas and ethnic minorities in the study, while drowning was the leading cause of death for urban areas and ethnic Han. In accordance with the characteristics that suffocation deaths mainly occur within 1 year old and in cold season as well as drowning deaths occur at 1 year old and above and summer, it’s necessary to pay special attention to the prevention of suffocation deaths in infants in winter and drowning deaths in older children in summer. We need to strengthen the prevention of suffocation in ethnic minority areas and the prevention of drowning in urban and Han areas. In addition, child deaths from poisoning, falls and traffic accidents have not changed much over the years. And with the rapid decline of drowning and suffocation deaths, the proportion of deaths from other causes must increase, and that,should be given equal attention, too.

## Conclusion

This study clarifies the timing, location, and demographic characteristics of children who died from unintentional injuries in Sichuan, making up for the lack of such a complete study in Sichuan. However, despite the large amount of data used in this study, we did not explore the causes in sufficient depth, such as the lack of analysis on emergency treatment measures and the lack of analysis on the construction of accidental injury prevention facilities. ​In the future, we should combine death surveillance with qualitative surveys or in-depth quantitative surveys to further provide evidence for reducing unintentional injury deaths in children.

## Data Availability

All data generated or analyzed during this study are included in this published article.
